# A Study on the Therapeutic Efficacy of San Zi Yang Qin Decoction for Non-Alcoholic Fatty Liver Disease and the Underlying Mechanism Based on Network Pharmacology

**DOI:** 10.1155/2021/8819245

**Published:** 2021-01-08

**Authors:** Yiping Li, Yang Liu, Ming Yang, Qianlei Wang, Yu Zheng, Jiaoya Xu, Peiyong Zheng, Haiyan Song

**Affiliations:** ^1^Institute of Digestive Diseases, Longhua Hospital, Shanghai University of Traditional Chinese Medicine, 725 Wanping Road, Shanghai 200032, China; ^2^Office of National Drug Clinical Trial, Longhua Hospital, Shanghai University of Traditional Chinese Medicine, Shanghai 200032, China; ^3^Department II of Digestive Diseases, Longhua Hospital, Shanghai University of Traditional Chinese Medicine, Shanghai 200120, China

## Abstract

**Objective:**

This study aims to explore the therapeutic efficacy of San Zi Yang Qin Decoction (SZ) and its potential mechanism in the treatment of non-alcoholic fatty liver disease (NAFLD) based on network pharmacology and *in vivo* experiments.

**Methods:**

Effective chemicals and targets of SZ were searched in online databases, according to the drug-likeness of compounds and the binomial distribution of targets. A disease-target-chemical network was established using NAFLD-associated genes screened through GeneCards database, Gene Ontology (GO) terms, and Kyoto Encyclopedia of Genes and Genomes (KEGG) pathways. Furthermore, animal experiments were conducted to verify the efficacy and mechanism of SZ predicted by network pharmacology. The NAFLD mouse model was established with C57BL/6J mice fed with a high-fat diet for 22 weeks. The mice in the control group were fed with a chow diet. From the 23^rd^ week, the NAFLD mice were treated with intragastric SZ or normal saline for 8 weeks. After the glucose tolerance was measured, the mice were sacrificed, followed by the collection of serum and liver tissues. Pathological changes in liver tissues were examined by H&E staining. Additionally, alanine aminotransferase (ALT), aspartate aminotransferase (AST), serum fast blood glucose, and insulin levels were detected. Expression levels of TNF-*α* of serum and liver tissues were determined by ELISA and qRT-PCR, respectively. Western blot was used to detect the activation of AKT in liver tissues.

**Results:**

A total of 27 effective compounds and 20 targets of SZ were screened. GO analysis uncovered a significant correlation between the targets of SZ and those of NAFLD. KEGG analysis presented the signaling pathways enriched in SZ and NAFLD, including NAFLD, TNF-*α*, and apoptosis pathways. The area under the curve of major GO and KEGG pathways indicated the potential role of SZ in improving NAFLD. *In vivo* experiments demonstrated that SZ significantly alleviated hepatosteatosis and inflammatory cell infiltration in liver tissues, reduced serum transaminases, and improved insulin resistance and glucose tolerance of NAFLD mice. The protein level of phospho-AKT was upregulated by SZ. Additionally, SZ treatment obviously impaired the TNF-*α* level in the serum and liver tissue of NAFLD mice.

**Conclusions:**

According to the network pharmacology analysis and *in vivo* experiments, SZ could have therapeutic efficacy for NALFD. The mechanism mainly involves pathways relative to insulin resistance, TNF-*α*, and apoptosis. Our results provide a scientific basis for SZ in the clinical treatment of NAFLD.

## 1. Introduction

Non-alcoholic fatty liver disease (NAFLD) is a syndrome of hepatic excessive fat deposition that does not arise from alcohol abuse or other definite factors [1]. The spectrum of NAFLD includes non-alcoholic fatty liver (NAFL), non-alcoholic steatohepatitis (NASH), liver cirrhosis, and hepatocellular carcinoma. NAFLD is the most common liver disorder, with an incidence of about 20–33% in adults worldwide and up to 75% in the obese [2]. About 20% of NAFLD cases progress to NASH with liver inflammation and fibrosis, and even refractory liver diseases like cirrhosis and liver cancer [3]. It is believed that NAFLD develops through a multi-hit process. At first, excessive deposition of fatty acids leads to hepatosteatosis. As systematic insulin sensitivity decreases, lipolysis initiates in adipose tissues and de novo fatty acid increases in the liver, further aggravating hepatic lipid accumulation. With the aggravation of NAFLD, lipotoxicity gradually induces endoplasmic reticulum stress, oxidative stress, mitochondrial dysfunction, and endotoxin-induced release of inflammatory cytokines, etc. Eventually, the multi-hit process causes hepatocyte apoptosis, liver inflammation, and fibrosis [4].

Due to its complex pathogenesis, NAFLD still lacks effective treatments. In recent years, TCM has been widely applied in the treatment of NAFLD in China, with a definite efficacy featuring holistic treatment and multi-target regulation [5]. From the perspective of TCM theory, NAFLD is defined as a disease of “obesity, phlegm-fluid retention, and accumulation,” according to its clinical symptoms and signs [6]. In NAFLD, the dysfunction of the liver in regulating Qi flow and the spleen in transforming and transporting food essence contribute to the pathological accumulation of phlegm, dampness, and stagnation of Qi.

San Zi Yang Qin Decoction (SZ), initially reported in Han Shi Yi Tong, is composed of Raphani semen (Laifuzi in China), Perilla fructus (Zisuzi in China), and Sinapis semen (Baijiezi in China). Raphani semen can reduce food stagnation and monitor Qi to eliminate phlegm. Perilla fructus can descend Qi to carry away phlegm, smooth the bowel, and relieve cough and asthma [7]. Sinapis semen is acrid and warm, facilitates Qi, dissipates gatherings, and resolves phlegm. Their combination can treat diseases resulting from abnormal Qi flow and phlegm stasis, like cough, short breath, excessive phlegm, chest impediment, and lack of appetite. SZ is particularly effective for pulmonary diseases, such as intractable cough, chronic obstructive pneumonia disease (COPD), and chronic bronchitis [8, 9]. According to the TCM theory of “treatment based on syndrome differentiation,” and “homotherapy for heteropathy” during which the same therapy is administered for different diseases sharing the same pathogenesis, SZ has been recently applied in the treatment of diseases resulting from the stagnation of phlegm, dampness, and Qi, like NAFLD, hyperlipidemia, type 2 diabetes mellitus (T2DM), and breast hyperplasia [10–15]. However, *in vitro* and *in vivo* experiments and evidence-based analyses have not been carried out to validate the efficacy and interpret the related biological mechanisms of SZ [16, 17].

Network pharmacology is a novel research model based on systems biology and bioinformatics theories. It seeks to understand how drugs work from the system perspective and on the molecular level [18, 19]. Diverse active ingredients in TCM, especially TCM formulae, demonstrate complicated interactions, thus posing a great challenge to the research on TCM pharmacology. Accumulating evidence can reveal the efficacy and the underlying mechanism of a single active ingredient extracted from Chinese herbal medicine but is far from enough to explain those of the formulae, and the interactions between ingredients [20]. Network pharmacology has revolutionized TCM research from “one ingredient and one target” to “a complex of ingredients and a network of targets.” Network pharmacology intends to illustrate the mechanism of a formula based on a systemic analysis [21, 22] and assess its possible efficacy according to the correlation between one chemical and its targets [23].

Using the approach of network pharmacology, the present study aimed to screen out the effective chemicals in SZ, define their targets associated with NAFLD, and describe the underlying mechanisms using online databases. Furthermore, we established an *in vivo* NAFLD mouse model to validate our results from database analyses.

## 2. Materials and Methods

### 2.1. Isolation of Main Chemicals in SZ

SZ is composed of Raphani semen, Perilla fructus, and Sinapis semen. The main chemicals in SZ were searched using the Traditional Chinese Medicine Systems Pharmacology Database and Analysis Platform (TCMSP, Version2.3) [24], Herb Ingredients' Targets (HIT) [25], Traditional Chinese Medicines Integrated Database (TCMID, Version2.0) [26], and Search Tool for Interactions of Chemical (STITCH, Version5.0) [27]. To estimate their drug-likeness, the physicochemical characteristics of collected chemicals were compared with those of known drugs, according to their AMDE (absorption, metabolism, distribution, excretion). In 2012, Liang et al. proposed that quantitative estimate of drug-likeness (QED) [28,29], an evaluation index of drug-likeness, could effectively estimate the AMDE of chemicals. QED was calculated using the following equation:

(1) $$\begin{array}{c} \text{QED }=\;\exp\left(\frac{1}{n}\sum_{i=1}^{n}\ln\;d_{i}\right). \end{array}$$

In this equation, *d*_*i*_ denotes the desirability function of 8 major molecular descriptors, including the relative molecular weight of the compound, oil/water partition coefficient, the number of hydrogen bond receptors, the number of hydrogen bond donors, polar surface area, the number of rotatable bonds in the compound, the number of aromatic rings in the compound, and the number of nonpharmaceutical substructures [30]. During the initial screening of chemicals, a threshold of 0.2 was set according to the QED of 1,805 drugs released by Food and Drug Administration (FDA) in the DrugBank [31].

### 2.2. Screening of Main Targets of Chemicals in SZ

Potential targets of the above-screened chemicals were searched from HIT, TCMID, STITCH, and TCMSP, and their names were normalized with the National Center for Biotechnology Information (NCBI) database. Enrichment score or gene score (GS) was calculated based on the binomial distribution [*p*(*X* ≥ *k*)] to screen out the targets:

(2) $$\begin{array}{c} p\left(X\geq k\right)=\sum_{m=k}^{k}C_{n}^{m}{\left(\frac{g}{n}\right)}^{m}{\left(1-\frac{g}{n}\right)}^{n-m}, \end{array}$$

(3) $$\begin{array}{c} \text{GS}=\left\{\begin{array}{cc} \frac{-\log\left(p\left(X\geq k\right)\right)}{\text{Rank}\left(p\left(X\geq k\right)\right)}, & \text{if }p\left(X\geq k\right)\leq 0.05,\\ 0, & \text{otherwise,} \end{array}\right\}, \end{array}$$

*k* is the number of chemicals with function on the studied targets, *n* is the total number of chemicals, and *g* is the average number of chemicals functioning on each target. After adjustment of the false discovery rate (FDR), the possibility of interaction among more than *k* chemicals in the total chemicals (*n*) of the targets was randomly calculated by equation (2). Equations (2) and (3) were used to assess the likelihood of random results. *p* < 0.05 suggested that the number of interactive chemicals was significantly larger than the expected and could be considered as the target goal. The database of SZ targets was established with an associated threshold of 400 and a significant threshold of 0.05.

### 2.3. Screening of Targets Associated with NAFLD

The relevant targets of NAFLD were searched from GeneCards [32] and funneled into a database.

### 2.4. Enrichment Analyses of GO and KEGG Pathways

Targets of SZ and NAFLD were subjected to enrichment analyses of Gene Ontology (GO) terms (including molecular functions, biological processes, and cellular components) and Kyoto Encyclopedia of Genes and Genomes (KEGG) pathways. A significant correlation (*p* < 0.01) between a gene set and GO terms and KEGG pathways was estimated by a hypergeometric distribution model as follows [23]:

(4) $$\begin{array}{c} p=1-\sum_{i=0}^{k-1}\frac{\left(\begin{array}{c} M\\ i \end{array}\right)\left(\begin{array}{c} N-M\\ n-i \end{array}\right)}{\left(\begin{array}{c} N\\ n \end{array}\right)}, \end{array}$$

where *N* is the total number of genes, *M* is the number of KEGG pathways or annotated GO terms, *n* is the number of genes in the gene set to be analyzed, and *k* is the number of shared genes. *p* value after adjustment of FDR [33] was used to reflect the association strength between the target and enriched pathways or GO terms. *p* < 0.01 was considered statistically significant.

### 2.5. Establishment of NAFLD Mouse Model and Drug Intervention

A total of 18 five-week-old male C57BL/6J mice (Shanghai SLAC Laboratory Animal Co., Ltd.) were housed in the SPF-level animal center of Longhua Hospital, Shanghai University of Traditional Chinese Medicine. The mice were randomly divided into the control group (*n* = 6) and NAFLD group (*n* = 12) and fed with a normal diet and a high-fat diet, respectively. From the 23^rd^ week, the mice in NAFLD group were randomly subgrouped into model group (*n* = 6) and SZ group (*n* = 6). The mice in SZ group were intervened by intragastric administration of SZ for eight weeks, while the remaining with normal saline. At the end of the experiment, all animals were fasted overnight and anesthetized through intraperitoneal injection of 30 mg/kg pentobarbital sodium. Blood samples were collected and serum was separated through centrifugation. The livers were collected and weighed and then frozen or fixed in 10% formalin for further investigation.

SZ, provided by the Pharmacy Department of Longhua Hospital, Shanghai University of Traditional Chinese Medicine, was composed of Raphani semen (9 g), Perilla fructus (9 g), and Sinapis semen (9 g). According to the guideline proposed by the Methodology of Pharmacology of Traditional Chinese Medicine, the dosage of SZ for each mouse was calculated to be equal to the clinical dose of a 70 kg adult (3.51 g crude drug/kg/d). These three herbs were mixed at a mass ratio of 1 : 1 : 1 and grinded and then extracted by boiling water twice (30 min each time). Afterwards, the total extract was concentrated to 3.51 g crude drug/mL and stored at 4°C. When used, each mouse was given 0.2 mL SZ at the corresponding concentration adjusted with 0.9% saline by gavage. The experimental protocols were approved by the Institutional Animal Care and Use Committee of Shanghai University of TCM (IACUC No.: LHERAW-19052).

### 2.6. Pathological Examination of Mouse Liver Tissues

After sacrifice, mouse liver tissues were fixed in 4% neutral paraformaldehyde solution for 24 h and then dehydrated and paraffin-embedded, until they were sliced into 5 *μ*m sections for H&E staining and observation.

### 2.7. Serum Biochemistry

Serum samples collected from mice were examined in the Laboratory of the Longhua Hospital, Shanghai University of Traditional Chinese Medicine. In brief, relative levels of alanine aminotransferase (ALT), aspartate aminotransferase (AST), and fasting blood glucose (FBG) were detected using an automatic biochemical analyzer with commercial reagents (Roche).

### 2.8. Intraperitoneal-Injected Glucose Tolerance Test (IPGTT)

IPGTT in the mice was conducted at the 8^th^ week of intragastric administration. After fasting for 12 h, the blood glucose (BG) level was first measured as a baseline. Subsequently, the mice were intraperitoneally administrated with 1 g/kg glucose, followed by determination of tail vein blood glucose at 15, 30, 60, and 90 min with an automated glucometer (Johnson, Shanghai, China). The area under the curve (AUC) was calculated for the results of IPGTT.

### 2.9. Western Blotting Analysis

The protein was extracted from liver tissues. BCA protein assay kit (CoWin Bioscience, Beijing, China) was applied to determine protein concentration. The protein (100 *μ*g for each sample) was separated with 10% sodium dodecyl sulfate-polyacrylamide gel electrophoresis (SDS-PAGE) and then transferred to a PVDF membrane (Millipore, Billerica, MA, USA). After blockade with 5% skim milk, the membrane was incubated with targeted primary antibodies overnight at 4°C and then with secondary antibody at room temperature for 1 h. The Phospho-AKT and *β*-actin antibodies and secondary antibodies were purchased from Cell Signaling Technology (Danvers, MA, USA). Finally, the signals were detected by enhanced chemiluminescence (ECL) Detection Kit (Millipore, Billerica, MA, USA) and captured by G: BOX Chemi XT4 System (Syngene, Cambridge, UK). GeneTools software (Syngene) was used for quantification. The relative protein expression was normalized to *β*-actin.

### 2.10. ELISA of Serum TNF-*α* and Insulin

Serum levels of TNF-*α* and fast insulin (FIN) of mice were detected using ELISA (enzyme-linked immunosorbent assay) according to the manufacturer's protocols labeled on commercial kits (Shanghai WestTang Bio-tech Co., Ltd.).

### 2.11. Calculation of HOMA-IR

The homeostasis model assessment of insulin resistance (HOMA-IR) is an index that quantifies insulin resistance. Mouse HOMA-IR was calculated as follows: HOMA-IR = FBG (mmol/L) × FINS (*μ*U/mL)/22.5. The unit conversion for FIN is as follows: 1 *μ*g/L = 1000/45.4 *μ*U/mL.

### 2.12. Quantitative RT-PCR

Primer sequences used in RT-PCR were verified using the Basic Local Alignment Search Tool (BLAST) and synthesized by Shanghai Shinegene Bio-tech Co., Ltd. (Table 1). RNA was isolated from mouse liver tissues using TRIzol method and reversely transcribed into cDNAs using the ABI reverse transcription kit. Later, cDNA was amplified by the StepOnePlus™ qRT-PCR system, with *β*-actin as the internal reference. Relative levels were calculated by 2^−ΔΔCT^method.

### 2.13. Statistical Analyses

Statistical analyses and figure formatting were conducted by using SPSS 24.0 and Graphpad Prism 7.0, respectively. Data were expressed as mean ± SD. Normally distributed data with equal variance were compared by one-way ANOVA, followed by LSD *t*-test. *p* < 0.05 was considered statistically significant.

## 3. Results

### 3.1. Effective Chemicals in SZ and Their Targets

According to the searching results from TCMSP, HIT, and TCMID, 52 types of chemicals were found in Raphani semen, 128 in Perilla fructus, and 51 in Sinapis semen. After calculation with equation (1) and QED threshold 0.2, 27 effective chemicals in SZ were obtained, mainly including *β*-sitosterol, luteolin, stigmasterol, and mustard oil, etc. (Table 2). A database of chemicals within SZ was then established.

### 3.2. Targets of Effective Chemicals in SZ

Potential targets of the above-screened chemicals were searched in HIT, TCMID, STITCH, and TCMSP and normalized using NCBI database. After the calculation of GS using equations (2) and (3), 20 targets were selected, mainly including protein kinase B1 (AKT1), tumor necrosis factor (TNF), and caspase-3 (CASP3) (Table 3). A database was then established based on these targets.

### 3.3. Screening Targets of NAFLD

By searching the keyword “non-alcoholic fatty liver disease” in GeneCards, 75 genes associated with human NAFLD and a correlation score >30 were selected (Table 4). A database about the target genes of NAFLD was then established.

### 3.4. GO Enrichment Analysis on Targets of SZ and NAFLD

**Figure 1:**
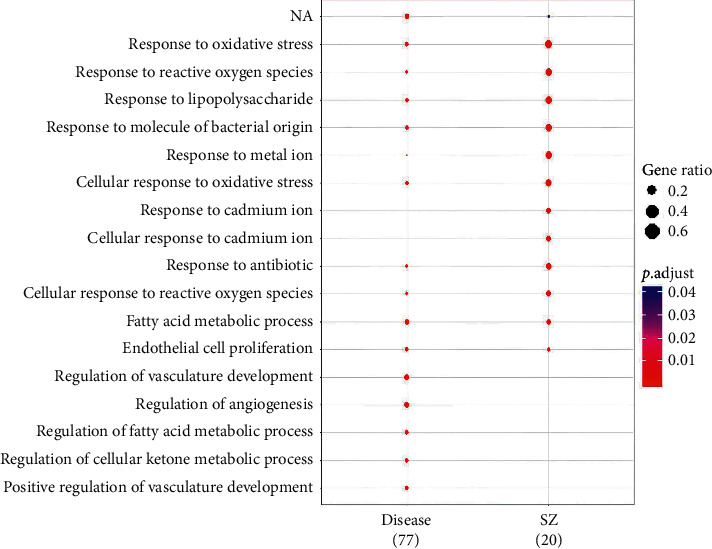
GO enrichment analysis of biological processes of SZ and NAFLD.

**Figure 2:**
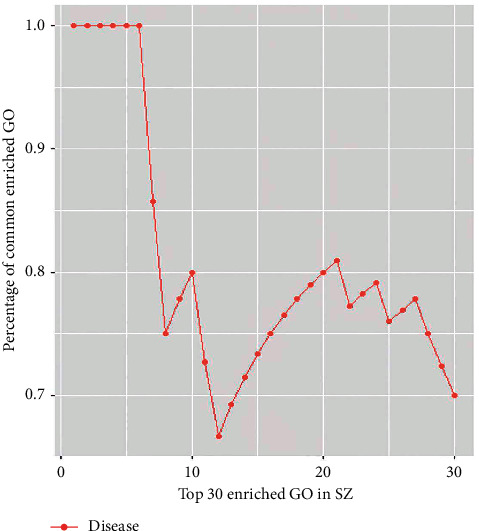
The cumulative distribution of percentages of common terms from the top 30 enriched GO terms in SZ for NAFLD.

A total of 20 main targets of SZ and 75 targets of NAFLD were subjected to GO enrichment analysis of molecular functions, biological processes, and cellular components. As shown in Figure 1 and Figures S1 and S2, targets of SZ and NAFLD were correlated with each other and mainly enriched in oxidative stress, lipopolysaccharide (LPS) response, and fatty acid metabolism. In addition, it is considered that the cumulative distribution percentages of common enriched targets or pathways can be used to reflect the correlation between the medicine and the disease. A disease that is more closely associated with a medicine shares more common targets or pathways in the top *k* enriched targets [23]. In the present study, the cumulative distribution of enriched GO targets was 38.93%, and the area under the curve (AUC) of the top 30 enriched GO targets was up to 23.388, suggesting a close correlation between targets of SZ and NAFLD (Figure 2).

### 3.5. KEGG Enrichment Analysis

**Figure 3:**
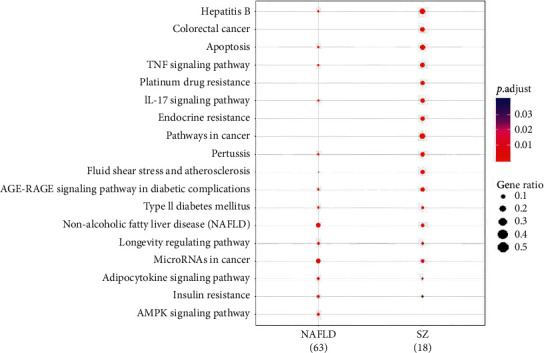
KEGG pathways enriched of SZ and NAFLD.

**Figure 4:**
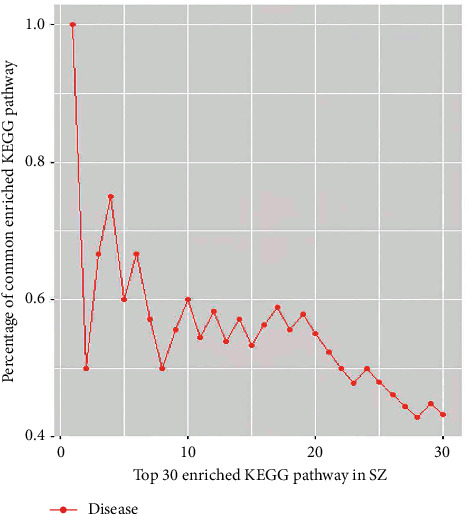
The cumulative distribution of percentages of common terms from the top 30 enriched KEGG terms in SZ for NAFLD.

Potential pathways enriched in the targets of SZ and NAFLD were explored by KEGG enrichment analysis. A total of 126 pathways were enriched in SZ targets, 46 in NAFLD targets, and 34 in both. A total of 18 top pathways are depicted in Figure 3, including NAFLD, TNF-*α*, apoptosis, T2DM, lifespan regulation, adipokines, insulin resistance pathway, etc. The pathways shared by SZ and NAFLD showed a similarity of up to 73.91%. In addition, the area under the curve of the top 30 overlapped pathways between SZ and NAFLD was 16.00 (Figure 4). These results showed multiple pathways were enriched in the targets of both SZ and NAFLD, suggesting a strong potential therapeutic efficacy of SZ on NAFLD.

**Figure 5:**
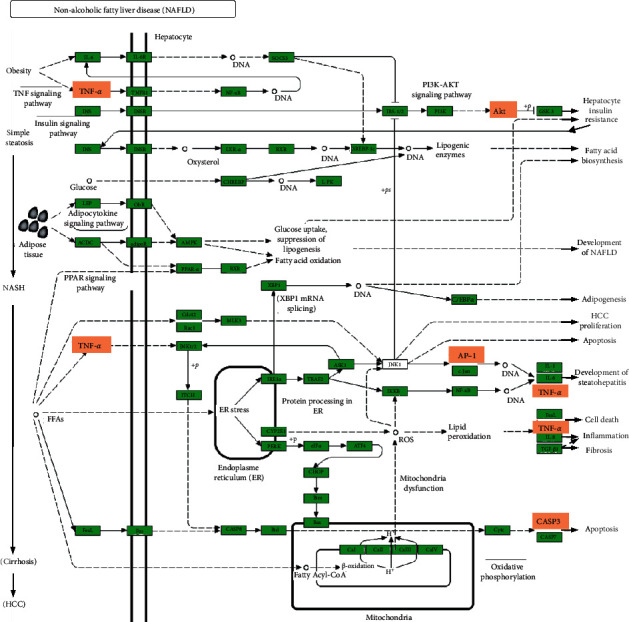
The NAFLD pathway enriched according to both SZ and NAFLD targets. The orange box marks the potential targets of SZ.

In Figure 5, the network of NAFLD pathways enriched in both SZ and NAFLD targets was depicted. The major molecular targets regulated by SZ were labeled with the red star, including TNF-*α*, AKT, activator protein 1 (AP-1), and caspase-3. They could promote the development and aggravation of NAFLD from the initial phase (excessive lipid accumulation) to NASH, through regulating insulin resistance, endoplasmic reticulum stress, oxidative stress, inflammatory response, fibrosis, and cell apoptosis. Taken together, SZ might counter NAFLD by regulating the above-searched pathways.

### 3.6. SZ Alleviated Liver Histopathological Changes and Improved Liver Function

**Figure 6:**
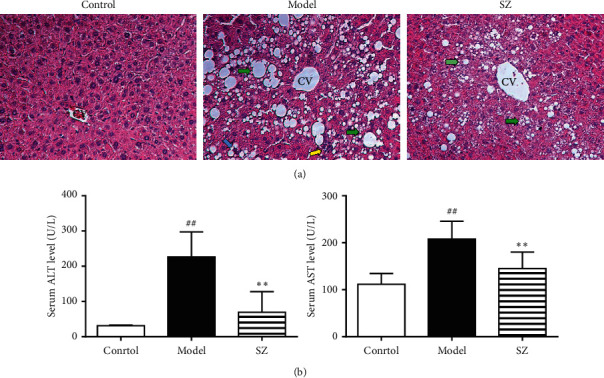
SZ alleviated liver tissue damage and improved liver function in NAFLD mice. (a) Representative photomicrographs of liver sections stained with hematoxylin and eosin (magnification: 200×). CV means centrilobular vein. The green arrow shows macrovesicular steatosis, the blue arrow shows ballooning degeneration of hepatocytes, and the yellow arrow shows infiltration of inflammatory cells. (b) Serum transaminase level. *n* = 6. ^##^*p* < 0.01 vs control; ^*∗∗*^*p* < 0.01 vs model.

H&E staining of liver tissues showed a normal structure of the hepatic lobules in control mice. NAFLD mice showed diffusely distributed macrovesicular and vesicular steatosis in the hepatic lobule, local infiltration of inflammatory cells, and ballooning degeneration of hepatocytes. In comparison to the model mice, the liver pathology was significantly alleviated after SZ treatment, as manifested by reduced area and severity of steatosis. Notably, local infiltration of inflammatory cells and ballooning degeneration were absent in SZ group (Figure 6). In addition, serum ALT and AST were significantly higher in the model group than in the control group (*p* < 0.01) and were remarkably reduced by SZ treatment (*p* < 0.01).

### 3.7. SZ Improved Glucose Tolerance and Insulin Resistance

**Figure 7:**
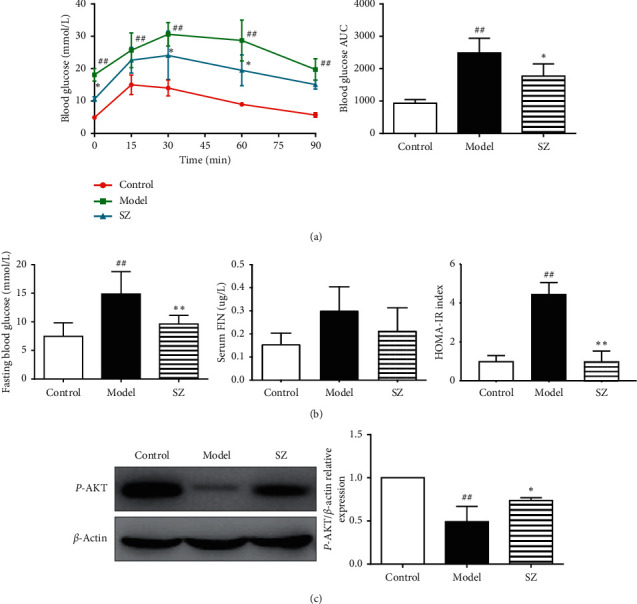
SZ increased glucose tolerance and decreased insulin resistance in NAFLD mice. (a) The IPGTT performed at the end of the 8-week treatment and the AUC calculated for the results of IPGTT. (b) Fasting blood glucose, insulin level, and the calculated HOMA-IR index. (c) The expression level of phospho-AKT in the liver tissues of mice. *n* = 4. ^#^*p* < 0.05 vs contro1; ^##^*p* < 0.01 vs control; ^*∗∗*^*p* < 0.01 vs model; ^*∗*^*p* < 0.05 vs model.

Compared with the mice in the control group, NAFLD mice showed significantly higher FBG (*p* < 0.01), which was reduced following SZ intervention (*p* < 0.01). IPGTT results and AUC values demonstrated that the ability of mice to regulate blood glucose was attenuated in the model group but obviously reversed by SZ treatment. HOMA-IR remained higher (*p* < 0.01) and phosphor-AKT was lower (*p* < 0.05) in NAFLD mice than in controls, indicating that insulin resistance existed in the model mice. SZ treatment significantly downregulated the HOMA-IR (*p* < 0.01) and activated hepatic AKT (*p* < 0.05) (Figure 7).

### 3.8. SZ Downregulated TNF-*α* Level of NAFLD Mice

**Figure 8:**
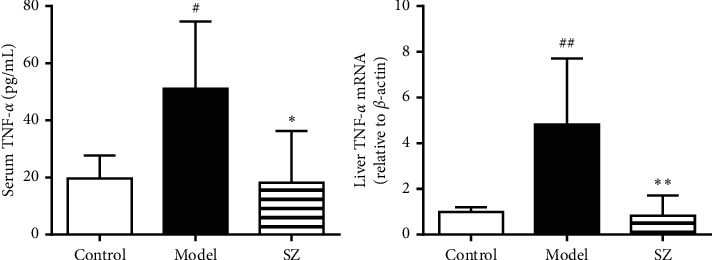
SZ downregulated TNF-*α* levels in serum and liver tissues of NAFLD mice. *n* = 6. ^#^*p* < 0.05 vs contro1; ^##^*p* < 0.01 vs control; ^*∗∗*^*p* < 0.01 vs model; ^*∗*^*p* < 0.05 vs model.

ELISA data revealed a higher serum TNF-*α* in the model group than in the control group (*p* < 0.05). After intervention with SZ, serum TNF-*α* in NAFLD mice was remarkably reduced (*p* < 0.05). Consistently, SZ significantly downregulated the mRNA level of TNF-*α* in the liver tissues of NAFLD mice (*p* < 0.01) (Figure 8).

## 4. Discussion

In this study, effective chemicals in SZ, their targets, and pathways enriched in NAFLD were searched through online databases. Meanwhile, the efficacy of SZ in the treatment of NAFLD was estimated by the approach of network pharmacology. A total of 27 effective chemicals of SZ were obtained, including *β*-sitosterol, luteolin, stigmasterol, mustard oil, etc. Some of them, such as *β*-sitosterol and luteolin, had been isolated and identified before [34, 35]. Then, 20 main targets highly correlated with NAFLD were screened out from the online database, including TNF, AKT, caspase-3, etc. Furthermore, GO and KEGG analyses showed that SZ was capable of regulating multiple biological processes and pathways enriched in NAFLD, TNF-*α*, apoptosis, T2DM, lifespan regulation, adipokines, insulin resistance pathway, etc. In another network pharmacology analysis of SZ in the treatment of asthma, 22 effective chemicals were extracted, which were similar to our study. In addition, enrichment analysis suggested that the therapeutic mechanism of SZ in treating asthma might involve signaling pathways of PI3K-Akt, TNF, and hypoxia inducible factor-1 [36]. This suggests that the traditional formula exerts similar effects on the regulation of molecular signaling pathways, even for different diseases. According to the method of Yang M. et al., a disease that is more closely associated with a medicine shares more terms in the top *k* enriched GO terms or KEGG pathways [23]. In the present study, the relative larger area under the curve of the top 30 GO terms and KEGG pathways was obtained, suggesting a close correlation and a stronger effect of SZ on NAFLD.

The approach of network pharmacology is just based on in silico analysis. Therefore, to validate the results from network pharmacology analysis, an *in vivo* NAFLD model was established with mice fed on a high-fat diet with or without SZ treatment. Diets rich in fat, like 30%–75% of total calories derived from saturated fatty acids (±unsaturated fatty acids), have been proved useful to induce metabolic alterations and NAFLD. This model can be used to mimic the pathological and molecular alterations in humans with NAFLD [37]. In this study, the mice fed with 30-week HFD displayed hepatosteatosis with inflammatory infiltration and ballooning hepatocytes in liver tissues, elevated serum ALT and AST, and decreased glucose tolerance, indicating the presence of NAFLD. SZ treatment significantly ameliorated histopathological changes (e.g., steatosis and inflammatory infiltration), downregulated serum transaminases, and improved glucose tolerance. These results confirmed the strong association between SZ and NAFLD predicted by network pharmacology analysis but also verified the effect of SZ on NAFLD predicted according to TCM theory.

It has been reported that modified San Zi Yang Qin Decoction (SZ plus dodder and glossy privet) could reduce the levels of FBG and 2 h postprandial blood glucose and increase the glucose tolerance in type 2 diabetes model rats induced by high-fat diet and multiple low-dose streptozotocin injections [15], which is consistent with our results. In addition, natural compounds in SZ, such as luteolin, *β*-sitosterol, and stigmasterol, can counter NAFLD or NASH in rodents. Luteolin alleviates NASH through repressing inflammatory pathways and oxidative stress [38]. *β*-Sitosterol prevents macrovesicular steatosis induced by a high-fructose diet and the progression of NAFLD to steatohepatitis [39]. After a 17-week treatment with 0.4% stigmasterol and *β*-sitosterol, NAFLD was significantly alleviated in mice fed with a high-fat western-style diet (HFWD) [40]. These natural compounds might be the effective ingredients of SZ; therefore, these experimental data also support our findings of the therapeutic efficacy of SZ for NAFLD.

The pathogenesis of NAFLD is a parallel multi-hit process, including insulin sensitivity, oxidative stress, mitochondrial dysfunction, the release of inflammatory cytokines, etc. [4]. According to our network pharmacology analysis, SZ may function on NAFLD through regulating pathways associated with insulin resistance (IR), TNF-*α*, endoplasmic reticulum stress, oxidative stress, and hepatocyte apoptosis. Through these regulations, SZ rebalances lipid metabolism and protects against liver injury in NAFLD.

IR is a key step during the development of NAFLD. The inactivation of AKT signaling pathway is the key factor of IR [41, 42]. The liver is an important target organ of insulin. Under physiological conditions, insulin can stimulate glycogen synthesis, repress the activities of gluconeogenesis enzymes, reduce blood glucose, and promote adipogenesis by activating AKT signaling pathway in hepatocytes [43]. Once insulin resistance occurs, an abnormally activated PI3K/AKT signaling pathway facilitates gluconeogenesis in the liver [44]. In the meantime, abnormally elevated insulin increases the synthesis of lipids in hepatocytes and decreases the oxidation of fatty acids, thus leading to the disorder of glucose and lipid metabolisms [45, 46]. Our *in vivo* data indicated that SZ significantly reduced FBG level and HOMA-IR, increased glucose tolerance, and dissipated liver fat accumulation in NAFLD mice. The AKT signaling pathway, once inactivated, was then upregulated by SZ treatment. These results demonstrated that SZ could ameliorate insulin resistance, which is consistent with that in our network pharmacological analysis. As one downstream molecule of IRS-1, AKT can be activated by phosphorylated IRS-1. Similarly, modified SZ can increase tyrosine-phosphorylated insulin receptor substrate-1 (IRS-1) in the skeletal muscle of T2DM rats to relieve insulin resistance [15].

TNF-*α* is critical for the development of NAFLD. It is reported that hepatocyte steatosis can activate NF-*κ*B to trigger the production of TNF-*α* in NAFLD. On the one hand, TNF-*α* induces hepatocyte apoptosis by activating caspase and releasing cytochrome C to form apoptotic bodies [47, 48]. On the other hand, TNF-*α* can lead to hepatocyte necrosis through inducing mitochondrial dysfunction, production of reactive oxygen species (ROS), and lipid peroxidation [49, 50]. All these processes aggravate simple steatosis of the liver into irreversible NASH with inflammatory responses [51]. TNF-*α* is also involved in lipid metabolism. It stimulates the release of free fatty acid (FFA), which in turn promotes the release of TNF-*α* from macrophages, eventually enhancing lipid accumulation in the liver [52, 53]. By directly targeting the intracellular insulin signal transduction system, TNF-*α* can enhance insulin resistance through upregulating suppressor of cytokine signaling 3 (SOCS-3) [54] and inhibiting adiponectin activity [55]. Our results showed that TNF-*α* level in serum and liver tissues was higher in NAFLD mice than in controls but remarkably reduced by SZ intervention. The *in vivo* results were consistent with those of our network pharmacological analysis in that SZ may rely on the TNF-*α* signaling pathway to reverse NAFLD. It has been reported that SZ could treat bronchial asthma by blocking cysteinyl leukotrienes- (CysLTs-) mediated inflammatory pathways [56]. The combination of SZ with Erehen decoction has shown significant clinical efficacy in the treatment of acute exacerbation of COPD. It can significantly improve lung function and reduce inflammatory factors [57]. As one component of SZ, in silico, *in vitro*, *in vivo*, and clinical studies strongly suggest that luteolin exerts its function mainly depending on its anti-inflammatory activity. It can protect against non-alcoholic steatohepatitis in HFD induced NASH rats. TNF-*α* and other proinflammatory cytokines levels were decreased in the groups that received 50 and 100 mg/kg luteolin [38, 58].

During the development of NAFLD, oxidative stress, endoplasmic reticulum stress, mitochondrial dysfunction, and TNF-*α* signaling are all associated with the activation of c-Jun N-terminal kinase (JNK). They cooperate to trigger the damage of hepatocytes [59, 60]. AP-1 is a downstream molecule of JNK. The homodimer or heterodimer of AP-1, fabricated by C-Jun and C-Fos family members, copes with multiple stimuli by binding to DNA sequences of target genes. As our study revealed, AP-1 might be a target of SZ, indicating that SZ alleviates multi-hit liver injury via regulating the JNK pathway. The component of SZ luteolin can also inhibit LPS-induced IL-6 production by repressing the JNK signaling pathway and AP-1 activation in microglia, a process that mitigates neuroinflammation. Experiments have confirmed that, as the upstream regulator, luteolin can alter the transcriptional activation of NF-*κ*B and AP-1 signaling pathways [58, 61]. During the development of NASH, multiple stimuli evoke hepatocyte apoptosis. Hepatocyte apoptosis is either a vital feature or a key factor leading to the aggravation of NASH. It is reported that the severity and extent of hepatocyte apoptosis in liver biopsy samples of NASH patients exceed those in non-NASH cases. The activity of caspase-3 is positively correlated with the symptom severity and pathologic stage of NASH [62]. Our results uncovered that caspase-3 may be targeted, and the apoptosis pathway is regulated by SZ. But, these findings still require further validation.

## 5. Conclusions

We have speculated that the TCM formula SZ might be applied to treat NAFLD according to the TCM principle of “homotherapy for heteropathy” and previously reported clinical cases. Based on network pharmacology-based analysis and *in vivo* experiments in SZ-intervened NAFLD mice, we for the first time verified the efficacy of SZ in alleviating NAFLD, providing evidence for its clinical application. This study also confirmed the TCM conception of “homotherapy for heteropathy” from the network molecular level. Meanwhile, SZ may improve NAFLD through targeting AKT, TNF-*α*, AP-1, and caspase-3, and regulating pathways enriched in insulin resistance, TNF-*α*, apoptosis, T2DM, etc. These findings should be further clarified through *in vivo* and *in vitro* experiments in the future.

## Figures and Tables

**Table 1:** The primer sequences for quantitative RT-PCR.

Gene	Primer sequence (5′-3′)
m*β*-Actin	Forward: GAGACCTTCAACACCCCAGC Reverse: ATGTCACGCACGATTTCCC

mTNF-*α*	Forward: CCCTCCAGAAAAGACACCATG Reverse: CACCCCGAAGTTCAGTAGACAG

**Table 2:** Active compounds of SZ.

Pubchem CID	Compound	QED
6431151 222284 5280794 3314 16741 5971 14985 5280445 5280443 998 6782 8434 6184 5997 3026 493570 2346 240 1140 7500 8259 16666 5283335 2724159 7362 7127 253	(-)-cis-Beta-Elemene beta-sitosterol Stigmasterol Eugenol Phenethyl isothiocyanate Allyl isothiocyanate Vitamin E Luteolin Apigenin Phenylacetaldehyde Diisobutyl phthalate Ethylparaben Hexanal Cholesterol Dibutyl phthalate Riboflavin Benzyl isothiocyanate Benzaldehyde Toluene Ethylbenzene 2,2,2- Trichloroethanol l-Menthol 2-Nonenal (-)-Perillaldehyde Furfural Methyleugenol Bioepiderm	0.2169 0.2418 0.2243 0.5907 0.4236 0.3523 0.2430 0.4015 0.4928 0.4882 0.4696 0.6242 0.3489 0.2663 0.3671 0.2302 0.4282 0.4731 0.3119 0.3400 0.3704 0.4438 0.2376 0.3818 0.5598 0.5328 0.3858

**Table 3:** The target genes of active compounds of SZ.

Gene ID	Gene symbol
836 7124 207 2936 3725 8856 9407 1543 218 224 2353 2950 4129 4318 5594 5595 7157 842 847 8989	CASP3 TNF AKT1 GSR JUN NR1I2 TMPRSS11D CYP1A1 ALDH3A1 ALDH3A2 FOS GSTP1 MAOB MMP9 MAPK1 MAPK3 TP53 CASP9 CAT TRPA1

**Table 4:** The target genes of NAFLD.

Gene ID	Gene symbol
5465 4481 1376 3953 1401 5468 7099 2678 5728 3586 7040 1499 207 836 338 335 406913 348 406906 3458 3553 5265 847 355 3643 406986 7157 51085 1571 3667 406901 80339 1649 406919 56729 407035 407015 3172 407040 2147 7422 5599 6347 7124 406983 406984 3077 3576 407018 19 406991 3030 407004 1636 213 3630 407029 9971 10062 406932 6720 4023 3569 406952 3479 100380876 9370 4547 406921 3952 174 26503 5444 2168 2875	PPARA MSR1 CPT2 LEPR CRP PPARG TLR4 GGT1 PTEN IL10 TGFB1 CTNNB1 AKT1 CASP3 APOB APOA1 MIR126 APOE MIR122 IFNG IL1B SERPINA1 CAT FAS INSR MIR203A TP53 MLXIPL CYP2E1 IRS1 MIR107 PNPLA3 DDIT3 MIR130A RETN MIR31 MIR26A1 HNF4Amir 34A F2 VEGFA MAPK8 CCL2 TNF MIR200Amir 200B HFE CXCL8 MIR27A ABCA1 MIR21 HADHA MIR22 ACE ALB INS MIR30A NR1H4 NR1H3 MIR140 SREBF1 LPL IL6 MIR17 IGF1 NAFLD1 ADIPOQ MTTP MIR132 LEP AFP SLC17A5 PON1 FABP1 GPT

## Data Availability

The datasets used to support the findings in the current study are included within the article and supplementary materials.

## Supplementary Materials

Supplementary Materials Figure S1: GO enrichment analysis of molecular functions. Figure S2: GO enrichment analysis of cellular components.
