# Supplementary material for: A Study on the Therapeutic Efficacy of San Zi Yang Qin Decoction for Non-Alcoholic Fatty Liver Disease and the Underlying Mechanism Based on Network Pharmacology
Source: Evid Based Complement Alternat Med. 2021 Jan 8;2021:8819245. doi: 10.1155/2021/8819245 (PMC7810527; doi:10.1155/2021/8819245)
Supplement: Supplementary Materials — Figure S1: GO enrichment analysis of molecular functions. Figure S2: GO enrichment analysis of cellular components. [file 8819245.f1.docx]

**Supplementary Materials**


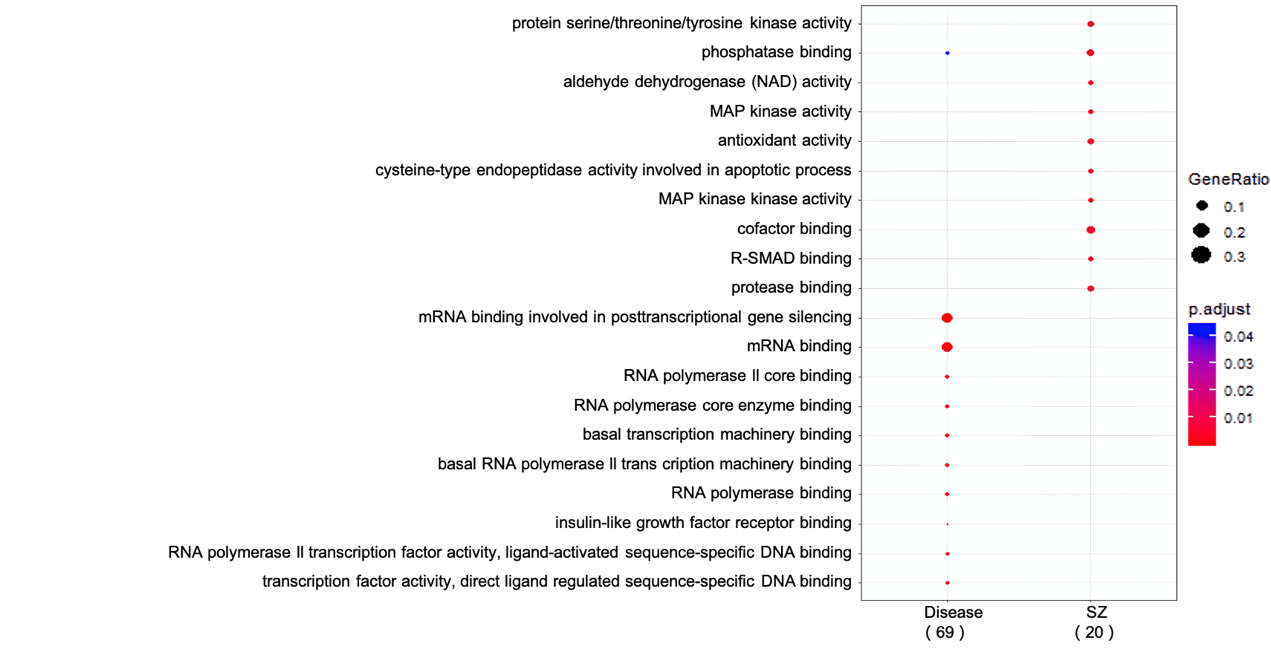


FIFURE S1: GO enrichment analysis of molecular functions.


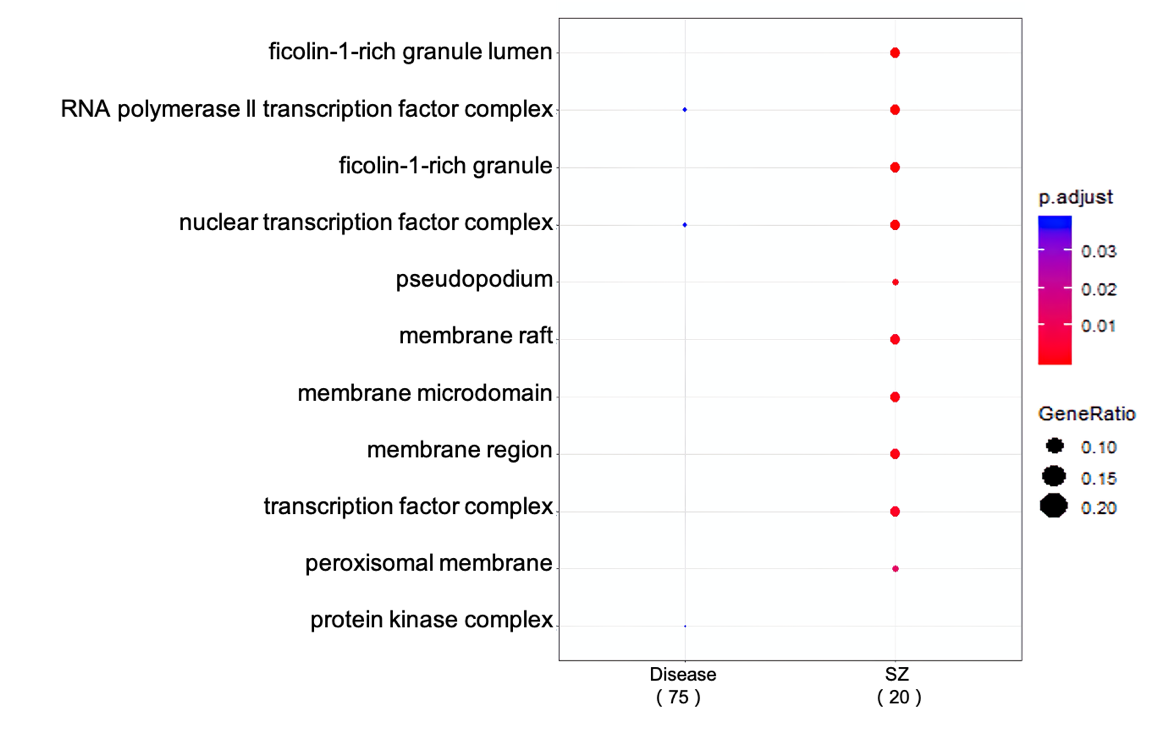


FIFURE S2: GO enrichment analysis of cellular components.
